# Partition function and base pairing probabilities of RNA heterodimers

**DOI:** 10.1186/1748-7188-1-3

**Published:** 2006-03-16

**Authors:** Stephan H Bernhart, Hakim Tafer, Ulrike Mückstein, Christoph Flamm, Peter F Stadler, Ivo L Hofacker

**Affiliations:** 1 Theoretical Biochemistry Group, Institute for Theoretical Chemistry, University of Vienna, Währingerstrasse 17, Vienna, Austria; 2 Bioinformatics Group, Department of Computer Science and Interdisciplinary Center for Bioinformatics, University of Leipzig, Härtelstrasse 16–18, D-04170 Leipzig, Germany; 3 The Santa Fe Institute, 1399 Hyde Park Rd., Santa Fe, New Mexico

## Abstract

**Background:**

RNA has been recognized as a key player in cellular regulation in recent years. In many cases, non-coding RNAs exert their function by binding to other nucleic acids, as in the case of microRNAs and snoRNAs. The specificity of these interactions derives from the stability of inter-molecular base pairing. The accurate computational treatment of RNA-RNA binding therefore lies at the heart of target prediction algorithms.

**Methods:**

The standard dynamic programming algorithms for computing secondary structures of linear single-stranded RNA molecules are extended to the co-folding of two interacting RNAs.

**Results:**

We present a program, RNAcofold, that computes the hybridization energy and base pairing pattern of a pair of interacting RNA molecules. In contrast to earlier approaches, complex internal structures in both RNAs are fully taken into account. RNAcofold supports the calculation of the minimum energy structure and of a complete set of suboptimal structures in an energy band above the ground state. Furthermore, it provides an extension of McCaskill's partition function algorithm to compute base pairing probabilities, realistic interaction energies, and equilibrium concentrations of duplex structures.

**Availability:**

RNAcofold is distributed as part of the Vienna RNA Package, .

**Contact:**

Stephan H. Bernhart – berni@tbi.univie.ac.at

## Background

Over the last decade, our picture of RNA as a mere information carrier has changed dramatically. Since the discovery of microRNAs and siRNAs (see e.g. [1,2] for a recent reviews), small noncoding RNAs have been recognized as key regulators in gene expression. Both computational surveys, e.g. [3-7] and experimental data [8-11] now provide compelling evidence that non-protein-coding transcripts are a common phenomenon. Indeed, at least in higher eukaryotes, the complexity of the non-coding RNome appears to be comparable with the complexity of the proteome. This extensive inventory of non-coding RNAs has been implicated in diverse mechanisms of gene regulation, see e.g. [12-16] for reviews.

Regulatory RNAs more often than not function by means of direct RNA-RNA binding. The specificity of these interactions is a direct consequence of complementary base pairing, allowing the same basic mechanisms to be used with very high specificity in large collections of target and effector RNAs. This mechanism underlies the post-transcriptional gene silencing pathways of microRNAs and siRNAs (reviewed e.g. in [17]), it is crucial for snoRNA-directed RNA editing [18], and it is used in the gRNA directed mRNA editing in kinetoplastids [19]. Furthermore, RNA-RNA interactions determine the specificity of important experimental techniques for changing the gene expression patterns including RNAi [20] and modifier RNAs [21-24].

RNA-RNA binding occurs by formation of stacked intermolecular base pairs, which of course compete with the propensity of both interacting partners to form intramolecular base pairs. These base pairing patterns, usually referred to as *secondary structures*, not only comprise the dominating part of the energetics of structure formation, they also appear as intermediates in the formation of the tertiary structure of RNAs [25], and they are in many cases well conserved in evolution. Consequently, secondary structures provide a convenient, and computationally tractable, approximation not only to RNA structure but also to the thermodynamics of RNA-RNA interaction.

From the computational point of view, this requires the extension of RNA folding algorithms to include intermolecular as well as intramolecular base pairs. Several approximations have been described in the literature: Rehmsmeier *et al. *[26] as well as Dimitrov and Zuker [27] introduced algorithms that consider exclusively intermolecular base pairs, leading to a drastic algorithmic simplification of the folding algorithms since multi-branch loops are by construction excluded in this case. Andronescu *et al. *[28], like the present contribution, consider all base pairs that can be formed in secondary structures in a concatenation of the two hybridizing molecules. This set in particular contains the complete structural ensemble of both partners in isolation. Mückstein *et al. *[29] recently considered an asymmetric model in which base pairing is unrestricted in a large target RNA, while the (short) interaction partner is restricted to intermolecular base pairs.

A consistent treatment of the thermodynamic aspects of RNA-RNA interactions requires that one takes into account the entire ensemble of suboptimal structures. This can be approximated by explicitly computing all structures in an energy band above the ground state. Corresponding algorithms are discussed in [30] for single RNAs and in [28] for two interacting RNAs. A more direct approach, that becomes much more efficient for larger molecules, is to directly compute the partition function of the entire ensemble along the lines of McCaskill's algorithm [31]. This is the main topic of the present contribution.

As pointed out by Dimitrov and Zuker [27], the concentration of the two interacting RNAs as well as the possibility to form homo-dimers plays an important role and cannot be neglected when quantitative predictions on RNA-RNA binding are required. In our implementation of RNAcofold we therefore follow their approach and explicitly compute the concentration dependencies of the equilibrium ensemble in a mixture of two partially hybridizing RNA species.

This contribution is organized as follows: We first review the energy model for RNA secondary structures and recall the minimum energy folding algorithm for simple linear RNA molecules. Then we discuss the modifications that are necessary to treat intermolecular base pairs in the partition function setting and describe the computation of base pairing probabilities. Then the equations for concentration dependencies are derived. Short sections summarize implementation, performance, as well as an application to real-world data.

## RNA secondary structures

A secondary structure *S *on a sequence *x *of length *n *is a set of base pairs (*i*, *j*), *i *<*j*, such that

0 (*i*, *j*) ∈ *S *implies that (*x*_*i*_, *x*_*j*_) is either a Watson-Crick (GC or AU) or a wobble (GU) base pair.

1 Every sequence position *i *takes part in at most one base pair, i.e., *S *is a matching in the graph of "legal" base pairs that can be formed within sequence *x*.

2 (*i*, *j*) ∈ *S *implies |*i *- *j*| ≥ 4, i.e., hairpin loops have at least three unpaired positions inside their closing pair.

3 If (*i*, *j*) ∈ *S *and (*k*, *l*) ∈ *S *with *i *<*k*, then either *i *<*j *<*k *<*l *or *i *<*k *<*l *<*j*. This condition rules out knots and pseudoknots. Together with condition 1 it implies that *S *is a circular matching [32,33].

The *"loops" *of *S *are planar faces of the unique planar embedding of the secondary structure graph (whose edges are the base pairs in *S *together with the backbone edges (*i*, *i *+ 1), *i *= 1 ..., *n *- 1). Equivalently, the loops are the elements of the unique minimum cycle basis of the secondary structure graph [34]. The *external loop *consists of all those nucleotides that are not enclosed by a base pair in *S*. The standard energy model for RNA secondary structures associates an energy contribution to each loop *L *that depends on the loop type type(*L*) (hairpin loop, interior loop, bulge, stacked pair, or multi-branch loop) and the sequence of some or all of the nucleotides in the loop, *x*|_*L*_:

*ε*(*L*) = *ε*(type(*L*), *x*|_*L*_).     (1)

The external loop does not contribute to the folding energy. The total energy of folding sequence *x *into a secondary structure *S *is then the sum over all loops of *S*. Energy parameters are available for both RNA [35] and single stranded DNA [36].

Hairpin loops are uniquely determined by their closing pair (*i*, *j*). The energy of a hairpin loop is tabulated in the form

(*i*, *j*) = (*x*_*i*_, *x*_*i*+1_, ℓ, *x*_*j*-1_, *x*_*j*_)     (2)

where ℓ is the length of the loop (expressed as the number of its unpaired nucleotides). Each interior loop is determined by the two base pairs enclosing it. Its energy is tabulated as

(*i*, *j*; *k*, *l*) = (*x*_*i*_, *x*_*i*+1_; ℓ_1_; *x*_*k*-1_, *x*_*k*_; *x*_*l*_, *x*_*l*+1_; ℓ_2_; *x*_*j*-1_, *x*_*j*_)     (3)

where ℓ_1 _is the length of the unpaired strand between *i *and *k *and ℓ_2 _is the length of the unpaired strand between *l *and *j*. Symmetry of the energy model dictates (*i*, *j*; *k*, *l*) = (*l*, *k*; *j*, *i*). If ℓ_1 _= ℓ_2 _= 0 we have a (stabilizing) stacked pair, if only one of ℓ_1 _and ℓ_2 _vanish we have a bulge. For multiloops, finally we have an additive energy model of the form  = *a *+ *b *× *β *+ *c *× ℓ where ℓ is the length of multiloop (again expressed as the number of unpaired nucleotides) and *β *is the number of branches, not counting the branch in which the closing pair of the loop resides.

So-called *dangling end *contributions arise from the stacking of unpaired bases to an adjacent base pair. We have to distinguish two types of dangling ends: (1) interior dangles, where the unpaired base *i *+ 1 stacks onto *i *of the adjacent basepair (*i*, *j*) and correspondingly *j *- 1 stacks onto *j *and (2) exterior dangles, where *i *- 1 stack onto *i *and *j *+ 1 stacks on *j*. The corresponding energy contributions are denoted by  and , respectively. Within the additive energy model, dangling end terms are interpreted as the contribution of 3' and 5' dangling nucleotides:



Here | separates the dangling nucleotide position from the adjacent base pair, *d*^5' ^(*k *- 1|*k*, *l*) thus is the energy of the nucleotide at position *k *- 1 when interacting with following base pair (*k*, *l*), while *d*^3' ^(*k*, *l*|*l *+ 1) scores the interaction of position *l *+ 1 with the preceding pair (*k*, *l*).

The Vienna RNA Package currently implements three different models for handling the dangling-end contributions: They can be (a) ignored, (b) taken into account for every combination of adjacent bases and base pairs, or (c) a more complex model can be used in which the unpaired base can stack with at most one base pair. In cases (a) and (b) one can absorb the dangling end contributions in the loop energies (with the exception of contributions in the external loop). Model (c) strictly speaking violates the secondary structure model in that an unpaired base *x*_*i *_between two base pairs (*x*_*p*_, *x*_*i*-1_) and (*x*_*i*+1_, *x*_*q*_) has three distinct states with different energies: *x*_*i *_does not stack to its neighbors, *x*_*i *_stacks to *x*_*i*-1_, or *x*_*i*+1_. The algorithm then minimizes over these possibilities. While model (c) is the default for computing minimum free energy structures in most implementations such as RNAfold and mfold, it is not tractable in a partition function approach in a consistent way unless different positions of the dangling ends are explicitly treated as different configurations.

## RNA secondary structure prediction

Because of the no-(pseudo)knot condition 3 above, every base pair (*i*, *j*) subdivides a secondary structure into an interior and an exterior structure that do not interact with each other. This observation is the starting point of all dynamic programming approaches to RNA folding, see e.g. [32,33,37]. Including various classes of pseudoknots is feasible in dynamic programming approaches [38-40] at the expense of a dramatic increase in computational costs, which precludes the application of these approaches to large molecules such as most mRNAs.

In the course of the "normal" RNA folding algorithm for linear RNA molecules as implemented in the Vienna RNA Package [41,42], and in a similar way in Michael Zuker's mfold package [43-45] the following arrays are computed for *i *<*j*:

*F*_*ij *_free energy of the optimal substructure on the subsequence *x*[*i*, *j*].

*C*_*ij *_free energy of the optimal substructure on the subsequence *x*[*i*, *j*] subject to the constraint that *i *and *j *form a basepair.

*M*_*ij *_free energy of the optimal substructure on the subsequence *x*[*i*, *j*] subject to the constraint that that *x*[*i*, *j*] is part of a multiloop and has at least one component, i.e., a sub-sequence that is enclosed by a base pair.

 free energy of the optimal substructure on the subsequence *x*[*i*, *j*] subject to the constraint that that *x*[*i*, *j*] is part of a multiloop and has exactly one component, which has the closing pair *i*, *h *for some *h *satisfying *i *≤ *h *<*j*.

The "conventional" energy minimization algorithm (for simplicity of presentation without dangling end contributions) for linear RNA molecules can be summarized in the following way, which corresponds to the recursions implemented in the Vienna RNA Package:



The *F *table is initialized as *F*_*i*+1, *i *_= 0, while the other tables are are set to infinity for empty intervals. It is straightforward to translate these recursions into recursions for the partition function because they already provide a partition of the set of all secondary structures that can be formed by the sequence *x*. This unambiguity of the decomposition of the ensemble structure is not important for energy minimization, while it is crucial for enumeration and hence also for the computation of the partition function [31]. Let us write *Z*_*ij *_for the partition function on [*x*_*i*_, *x*_*j*_].  for the partition function constrained to structures with an (*i*, *j*) pair, and ,  for the partition function versions of the multiloop terms *M*_*ij *_and .

The adaptation of the recursion to the folding of two RNAs *A *and *B *of length *n*_1 _and *n*_2 _into a dimeric structure is straightforward: the two molecules are concatenated to form a single sequence of length *n *= *n*_1 _+ *n*_2_. It follows from the algorithmic considerations below that the order of the two parts is arbitrary.

A basic limitation of this approach arises from the no-pseudoknots condition: It restricts not only the intramolecular base pairs but also affects intermolecular pairs. Let *S*^*A *^and *S*^*B *^denote the intramolecular pairs in a cofolded structure *S*. These sets of base pairs define secondary structures on *A *and *B *respectively. Because of the no-pseudoknot condition on *S*, an intermolecular base pair in *S*\(*S*^*A *^∪ *S*^*B*^) can only connect nucleotides in the external loops of *A *and *B*. This is a serious restriction for some applications, because it excludes among other pseudoknot-like structures also the so-called *kissing hairpin *complexes [46]. Taking such structures into account is equivalent to employing folding algorithms for structure models that include certain types of pseudoknots, such as the partition function approach by Dirks and Pierce [40]. Its high computational cost, however, precludes the analysis of large mRNAs. In an alternative model [29], no intramolecular interactions are allowed in the small partner *B*, thus allowing *B *to form basepairs with all contiguous unpaired regions in *S*^*A*^. From a biophysical point of view, however, it makes sense to consider exclusively hybridization in the exterior loop provided both partners are large structured RNAs. In this case, hybridization either stops early, i.e., at a kissing hairpin complex (in the case of very stable local structures) or it is thermodynamically controlled and runs into the ground state via a complete melting of the local structure. In the latter case, the no-pseudoknots condition is the same approximation that is also made when folding individual molecules. Note that this approximation does *not *imply that the process of hybridization could only *start *at external bases.

Let us now consider the algorithmic details of folding two concatenated RNA sequences. The missing backbone edge between the last nucleotide of the first molecule, position *n*_1 _in the concatenated sequence, and the first nucleotide of the second molecule (now numbered *n*_1_+1) will be referred to as the *cut c*. In each dimeric structure there is a unique loop *L*_*c *_that contains the cut *c*. If *c *lies in the external loop of a structure *S *then the two molecules *A *and *B *do no interact in this structure. Algorithmically, *L*_*c *_is either a hairpin loop, interior loop, or multibranch loop. From an energetic point of view, however, *L*_*c *_is an exterior loop, i.e., it does not contribute to the folding energy (relative to the random coil reference state). For example, an interior loop (*i*, *j*; *k*, *l*) does not contribute to the energy if either *i *≤ *n*_1 _<*k *or *l *≤ *n*_1 _<*j*. Naturally, dangling end contributions must not span the *cut*, either. Hairpin loops and interior loops (including the special cases of bulges and stacked pairs) can therefore be dealt with by a simple modification of the energy rules. In the case of the multiloop there is also no problem as long as one is only interested in energy minimization, since multiloops are always destabilizing and hence have strictly positive energy contribution. Such a modified MFE algorithm has been described already in [41].

**Figure 1:**
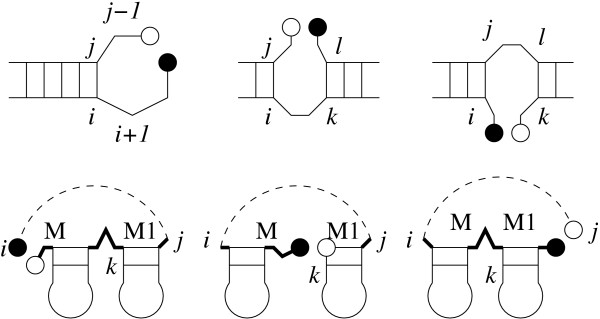
Loops with cuts have to be scored differently. Top row: hairpins and interior loops containing the cut between *n*_1 _(black ball) and *n*_1 _+ 1 (white ball). Below: multi loops containing the cut. Neither *M*1 nor *M *components must start at *n*_1 _+ 1 or stop at *n*_1_. Note that the construction of *Z*^*M *^out of *Z*^*M *^and *Z*^*M*1 ^ensures that the cut is not inside the loop part of *Z*^*M *^either.

For partition function calculations and the generation of suboptimal structures, however, we have to ensure that every secondary structure is counted exactly once. This requires one to explicitly keep track of loops that contain the cut *c*. The cut *c *needs to be taken into account explicitly only in the recursion for the *Z*^*P *^terms, where one has to distinguish between true hairpin and interior loops with closing pair (*i*, *j*) (upper alternatives in eq.(6)) and loops containing the cut *c *in their backbone (lower alternatives in eq.(6)). Explicitly, this means *i *≤ *n*_1 _<*j *in the hairpin loop case, in the interior loop case, this either means *i *≤ *n*_1 _<*k *or *l *≤ *n*_1 _<*j*. Since multiloops are decomposed into two components, it is sufficient to ensure during the construction of *Z*^*M*1 ^and *Z*^*M *^that these components neither start nor end adjacent to the cut, see Fig. 1.

In their full form including dangling end terms, the forward recursions for the partition function of an interacting pair of RNAs become



Upper alternatives refer to regular loops, lower alternatives to the loop containing the cutpoint. For brevity we have used here abbreviations (*i*, *j*) = exp(-(*i*, *j*)/*RT*), and equivalently , , , , , for the Boltzmann factors of the energy contributions. In the remainder of this presentation we will again suppress the dangling end terms for simplicity of presentation.

A second complication arises from the *initiation energy *Φ_*I *_that describes the entropy necessary to bring the two molecules into contact. This term, which is considered to be independent of sequence length and composition [47], has to be taken into account exactly once for every dimer structure if and only if the structure contains at least one base pair (*i*, *j*) that crosses the cut, i.e., *i *≤ *n*_1 _<*j*. The resulting bookkeeping problems fortunately can be avoided by introducing this term only after the dynamic programming tables have been filled. To this end we observe that *Z*_*i*, *j *_= , 1 ≤ *i*, *j *≤ *n*_1 _are the partition functions for subsequences of the isolated *A *molecule, while , 1 ≤ *i*, *j *≤ *n*_2 _are the corresponding quantities for the second interaction partner. Thus we can immediately compute the partition function *Z*^*AB *^- *Z*^*A*^*Z*^*B *^that counts only the structures with intermolecular pairs, i.e., those that carry the additional initiation energy contribution. The total partition function including the initiation term is therefore



## Base pairing probabilities

McCaskill's algorithm [31] computes the base pairing probabilities from the partition functions of subsequences. Again, it seems easier to first perform the backtracking recursions on the "raw" partition functions that do not take into account the initiation contribution. This yields pairing probabilities *P*_*kl *_for an ensemble of structures that does not distinguish between true dimers and isolated structures for *A *and *B *and ignores the initiation energy. McCaskill's backwards recursions are formally almost identical to the case of folding a single linear sequence. We only have to exclude multiloop contributions in which the cut-point *u *between components coincides with the cut point *c*. All other cases are already taken care of in the forward recursion.

Thus:



The "raw" values of *P*_*ij*_, which are computed without the initiation term, can now be corrected for this effect. To this end, we separately run the backward recursion starting from *Z*_1,*n *_and from  to obtain the base pairing probability matrices  and  for the isolated molecules. Note that equivalently we could compute  and  directly using the partition function version of RNAfold.

In solution, the probability of an intermolecular base pair is proportional to the (concentration dependent) probability that a dimer is formed at all. Thus, it makes sense to consider the conditional pair probabilities given that a dimer is formed, or not. The fraction of structures without intermolecular pairs in our partition function *Z *(i.e. in the cofold model without initiation contributions) is *Z*^*A*^*Z*^*B*^/*Z*, and hence the fraction of true dimers is



Now consider a base pair (*i*, *j*). If *i *∈ *A *and *j *∈ *B*, it must arise from the dimeric state. If *i*, *j *∈ *A *or *i*, *j *∈ *B*, however, it arises from the dimeric state with probability p* and from the monomeric state with probability 1 - p*. Thus the conditional pairing probabilities in the dimeric complexes can be computed as



The fraction of monomeric and dimeric structures, however, cannot be directly computed from the above model. As we shall see below, the solution of this problem requires that we explicitly take the concentrations of RNAs into account.

## Concentration dependence of RNA-RNA hybridization

Consider a (dilute) solution of two nucleic acid sequences *A *and *B *with concentrations *a *and *b*, respectively. Hybridization yields a distribution of five molecular species: the two monomers *A *and *B*, the two homodimers *AA *and *BB*, and the heterodimer *AB*. In principle, of course, more complex oligomers might also arise, we will, however, neglect them in our approach. We may argue that ternary and higher complexes are disfavored by additional destabilizing initiation entropies.

The presentation in this section closely follows a recent paper by Dimitrov [27], albeit we use here slightly different definitions of the partitions functions. The partition functions of the secondary structures of the monomeric states are *Z*^*A *^and *Z*^*B*^, respectively, as introduced in the previous section. In contrast to [27], we include the unfolded states in these partition functions. The partition functions *Z*^*AA*^, *Z*^*BB*^, and *Z*^*AB*^, which are the output of the RNAcofold algorithm (denoted *Z *in the previous section), include those states in which each monomer forms base-pairs only within itself as well as the unfolded monomers. We can now define



as the partition functions restricted to the true dimer states, but neglecting the initiation energies Θ_*I*_. An additional symmetry correction is needed in the case of the homo-dimers: A structure of a homo-dimer is symmetric if for any base pair (*i*, *j*) there exists a pair (*i'*, *j*'), where *i' *(*j'*) denotes the equivalent of position *i *in the other copy of the molecule. Such symmetric structures have a two-fold rotational symmetry that reduces their conformation space by a factor of 2, resulting in an entropic penalty of Δ*G*_*sym *_= *RT *ln 2. On the other hand, since the recursion for the partition functions eq. 6 assumes two distinguishable molecules *A *and *B*, any asymmetric structures of a homo-dimer are in fact counted twice by the recursion. Leading to the same correction as for symmetric structures.

Since both the initiation energy Θ_*I *_and the symmetry correction Δ*G*_*sym *_are independent of the sequence length and composition, the thermodynamically correct partition functions for the three dimer species are given by



From the partition functions we get the free energies of the dimer species, such as *F*^*AB *^= -*RT *ln , and the free energy of binding Δ*F *= *F*^*AB *^- *F*^*A *^- *F*^*B*^. We assume that pressure and volume are constant and that the solution is sufficiently dilute so that excluded volume effects can be neglected. The many particle partition function for this system is therefore [27]



where *a *= *n*_*A *_+ 2*n*_*AA*_+ *n*_*AB *_is the total number of molecules of type *A *put into the solution (equivalently for *b*); *n*_*A*_, *n*_*B*_, *n*_*AA*_, *n*_*BB*_, *n*_*AB *_are the particle numbers for the five different monomer and dimer species, *V *is the volume and *n *is the sum of the particle numbers. The system now minimizes the free energy -*kT *ln , i.e., it maximizes , by choosing the particle numbers optimally.

As in [27], the dimer concentrations are therefore determined by the mass action equilibria:

[*AA*] = *K*_*AA*_[*A*]^2^

[*BB*] = *K*_*BB*_[*B*]^2^

[*AB*] = *K*_*AB*_[*A*][*B*]     (14)

with



Concentrations in eq.(14) are in mol/l.

Note, however, that the equilibrium constants in eq.(15) are computed from a different microscopic model than in [27], which in particular also includes internal base pairs within the dimers.

Together with the constraints on particle numbers, eq.(14) forms a complete set of equations to determine *x *= [*A*] and *y *= [*B*] from *a *and *b *by solving the resulting quadratic equation in two variables:

0 = *f*(*x*, *y*) : = *x *+ *K*_*AB*_*xy *+ 2*K*_*AB*_*x*^2 ^- *a*

0 = *g*(*x*, *y*) : = *y *+ *K*_*AB*_*xy *+ 2*K*_*BB*_*y*^2 ^- *b *    (16)

The Jacobian



of this system is strictly positive and diagonally dominated, and hence invertible on ℝ^+ ^× ℝ^+^. Furthermore *f *and *g *are thrice continuously differentiable on  = [0, *a*] × [0, *b*] and we know (because of mass conservation and the finiteness of the equilibrium constants) that the solution (, ) is contained in the interior of the rectangle . Newton's iteration method



thus converges(at least) quadratically [48, 5.4.2]. We use (*a*, *b*) as initial values for the iteration.

## Implementation and performance

The algorithm is implemented in ANSI C, and is distributed as part of the of the *Vienna RNA *package. The resource requirements of RNAcofold and RNAfold are theoretically the same: both require (*n*^3^) CPU time and (*n*^2^) memory. In practice, however, keeping track of the cut makes the evaluation of the loop energies much more expensive and increases the CPU time requirements by an order of magnitude: RNAcofold takes about 22 minutes to *cofold *an about 3000 nt mRNA with a 20 nt miRNA on an Intel Pentium 4 (3.2 GHz), while RNAfold takes about 3 minutes to fold the concatenated molecule.

**Figure 2:**
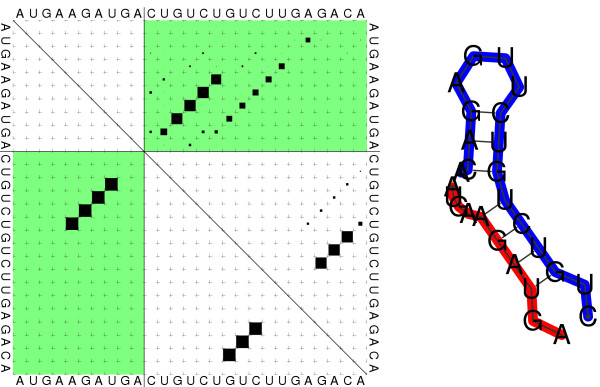
Dot plot (left) and mfe structure representation (right) of the cofolding structure of the two RNA molecules AUGAAGAUGA (red) and CUGUCUGUCUUGAGACA (blue). Dot Plot: Upper right: Partition function. The area of the squares is proportional to the corresponding pair probabilities. Lower left: Minimum free energy structure. The two lines forming a cross indicate the cut point, intermolecular base pairs are depicted in the green upper right (partition function) and lower left (mfe) rectangle.

**Figure 3:**
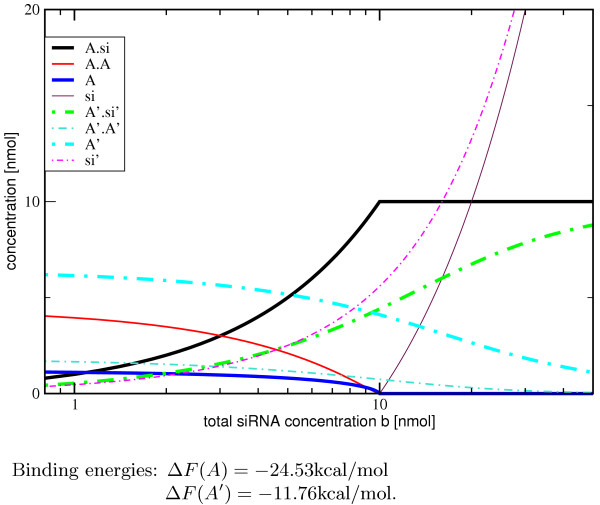
Example for the concentration dependency for two mRNA-siRNA binding experiments. In [54], Schubert *et al. *designed several mRNAs with identical target sites for an siRNA *si*, which are located in different secondary structures. In variant *A*, the *VR1 straight *mRNA, the binding site is unpaired, while in the mutant mRNA *VR1 HP5-11*, *A'*, only 11 bases remain unpaired. We assume an mRNA concentration of *a *= 10 nmol/1 for both experiments. Despite the similar binding pattern, the binding energies (Δ*F *= *F*^*AB *^- *F*^*A *^- *F*^*B*^) differ dramatically. In [54], the authors observed 10% expression for *VR1 straight*, and 30% expression for the HP5-11 mutant. Our calculation shows that even if siRNA is added in excess, a large fraction of the *VR1 HP5-11 *mRNA remains unbound.

The base pairing probabilities are represented as a *dot plot *in which squares with an area proportional to *P*_*ij *_represent the the raw pairing probabilities, see Fig. 2. The dot plot is provided as Postscript file which is structured in such a way that the raw data can be easily recovered explicitly. RNAcofold also computes a table of monomer and dimer concentrations dependent on a set of user supplied initial conditions. This feature can readily be used to investigate the concentration dependence of RNA-RNA hybridization, see Fig. 3 for an example.

Like RNAfold, RNAcofold can be used to compute *DNA dimers *by replacing the RNA parameter set by a suitable set of DNA parameters. At present, the computation of DNA-RNA heterodimers is not supported. This would not only require a complete set of DNA-RNA parameters (stacking energies are available [49], but we are not aware of a complete set of loop energies) but also further complicate the evaluation of the loop energy contributions since pure RNA and pure DNA loops will have to be distinguished from mixed RNA-DNA loops.

## Applications

Intermolecular binding of RNA molecules is important in a broad spectrum of cases, ranging from mRNA accessibility to siRNA or miRNA binding, RNA probe design, or designing RNA openers [50]. An important question that arises repeatedly is to explain differences in RNA-RNA binding between seemingly very similar or even identical binding sites. As demonstrated e.g. in [22,29,51,52], different RNA secondary structure of the target molecule can have dramatic effects on binding affinities even if the sequence of the binding site is identical.

**Figure 4:**
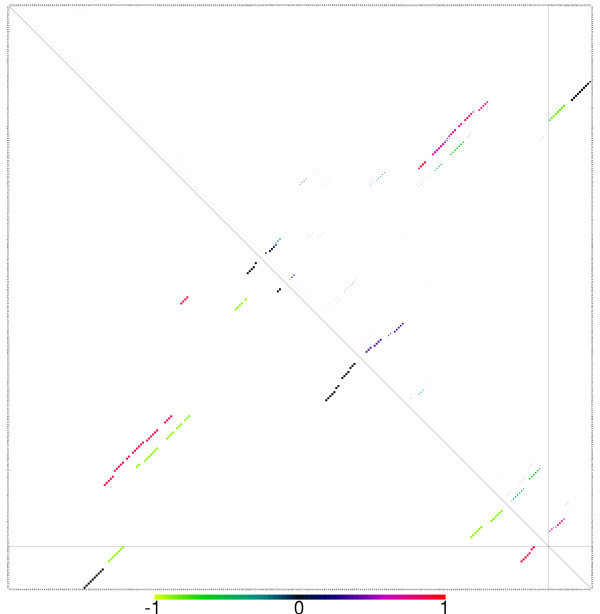
Difference dot Plot of native and mutated secondary structure of a 3 GU mutation of the CXCR4 siRNA gene. The red part on the right hand side shows the base pairing probability of the 5' part of the micro RNA, which is 80% higher in the native structure. This is an alternative explanation for the missing function of the mutant. Because of the mutations, the stack a little to the left gets more stable, and the probability of binding of the 5' end of the siRNA is reduced significantly.The color of the dots encodes the difference of the pair probabilities in the two molecules such positive (red) squares denote pairs more more probable in the second molecule (see color bar). The area of the dots is proportional to the larger of the two pair probabilities.

Since the comparison of base pairing patterns is a crucial step in such investigations we provide a tool for graphically comparing two dot plots, see Fig. 4. It is written in Perl-Tk and takes two *dot plot *files and, optionally, an alignment file as input. The differences between the two *dot plots *are displayed in color-code, the *dot plot *is zoomable and the identity and probability(-difference) of a base pair is displayed when a box is clicked.

As a simple example for the applicability of RNAcofold, we re-evaluate here parts of a recent study by Doench and Sharp [53]. In this work, the influence of GU base pairs on the effectivity of translation attenuation by miRNAs is assayed by mutating binding sites and comparing attenuation effectivity to wild type binding sites

Introducing three GU base pairs into the mRNA/miRNA duplex did, with only minor changes to the binding energy, almost completely destroy the functionality of the binding site. While Doench and Sharp concluded that miRNA binding sites are not functional because of the GU base pairs, testing the dimer with RNAcofold shows that there is also a significant difference in the cofolding structure that might account for the activity difference without invoking sequence specificities: Because of the secondary structure of the target, the binding at the 5' end of the miRNA is much weaker than in the wild type, Fig. 4.

## Limitations and future extensions

We have described here an algorithm to compute the partition function of the secondary structure of RNA dimers and to model in detail the thermodynamics of a mixture of two RNA species. At present, RNAcofold implements the most sophisticated method for modeling the interactions of two (large) RNAs. Because the no-pseudoknot condition is enforced to limit computational costs, our approach disregards certain interaction structures that are known to be important, including kissing hairpin complexes.

The second limitation, which is of potential importance in particular in histochemical applications, is the restriction to dimeric complexes. More complex oligomers are likely to form in reality. The generalization of the present approach to trimers or tetramers is complicated by the fact that for more than two molecules the results of the calculation are not independent of the order of the concatenation any more, so that for *M*-mers (*M *- 1)! permutations have to be considered separately. This also leads to bookkeeping problems since every secondary structure still has to be counted exactly once.
